# A pupillometric study of developmental and individual differences in cognitive effort in visual word recognition

**DOI:** 10.1038/s41598-022-14536-9

**Published:** 2022-06-24

**Authors:** Adi Shechter, Ronen Hershman, David L. Share

**Affiliations:** 1 Department of Learning Disabilities, Faculty of Education and Edmond J. Safra Brain Research Center for the Study of Learning Disabilities, University of Haifa, Haifa, Israel; 2 Department of Psychology and Zlotowski Center for Neuroscience, Ben-Gurion University of the Negev, Beer-Sheva, Israel

**Keywords:** Human behaviour, Neuroscience

## Abstract

Throughout the history of modern psychology, the neural basis of cognitive performance, and particularly its efficiency, has been assumed to be an essential determinant of developmental and individual differences in a wide range of human behaviors. Here, we examine one aspect of cognitive efficiency—cognitive effort, using pupillometry to examine differences in word reading among adults (*N* = 34) and children (*N* = 34). The developmental analyses confirmed that children invested more effort in reading than adults, as indicated by larger and sustained pupillary responses. The within-age (individual difference) analyses comparing faster (*N* = 10) and slower (*N* = 10) performers revealed that in both age groups, the faster readers demonstrated accelerated pupillary responses compared to slower readers, although both groups invested a similar overall degree of cognitive effort. These findings have the potential to open up new avenues of research in the study of skill growth in word recognition and many other domains of skill learning.

## Introduction

The general notion of individual differences in human performance has been around since the dawn of modern psychology^1^. One explanation for this diversity among individuals is the efficiency with which the nervous system functions when cognitive procedures are executed^2,3^. Increased efficiency is also assumed to underlie the developmental transition in skill learning from novice to expert^4^.

Cognitive efficiency is often described as the relationship between the quality of the observed performance and its costs, such as the cognitive effort invested^5,6^. Although cognitive efficiency is typically treated as a domain-general concept, it has been defined and operationalized somewhat differently by researchers in diverse fields^6,7^. Nonetheless, each conceptual approach shares the idea that cognitive effort is a core component of efficiency^5–8^.

Cognitive efficiency is also assumed to reflect neural activation. As far back as 1951, Landis suggested that one of the best indicators of efficiency is the *speed* with which the nervous system responds^9^. Recent studies have confirmed this assumption by showing that one aspect of individual differences in intelligence is neural processing speed^10^. More recently, Neubauer and Fink^11^ proposed their *neural efficiency theory* in which they operationalized efficiency as "the *amount* [emphasis added] of resources used to perform a given cognitive task correctly" (p. 1018). According to this theory, superior performers display less brain activation in order to perform a cognitive task successfully.

In accordance with the neural efficiency theory, a number of neuroimaging studies examining the relations between neural activation and reaction time among adults have reported that faster-performing individuals show less neural activity than slower performers^12–14^. These findings suggest that fast performance of cognitive tasks allows individuals to minimize their resource allocation while maximizing their performance^15^. However, Gray, Chabris, and Braver^16^ argued that this may be a faulty interpretation of these data. Superior performers may show stronger brain activation but for a shorter period of time, a pattern which is related to their faster performance. Consequently, they might demonstrate lower activation averaged over time compared to lower performing individuals. Indeed, in contrast to the neural efficiency theory, several investigations have shown that high-ability participants demonstrated greater neural activity than low-ability participants^16–18^.

Within the fields of neuroscience and psychology, cognitive efficiency has typically been measured by observing the neural activation patterns during task performance, as detected by brain-imaging techniques such as functional magnetic resonance imaging (fMRI), positron emission tomography (PET) and event-related potential (ERP)^6,11,19^. Another avenue for investigating cognitive efficiency and effort is pupillometry. While performing a cognitive task, the sympathetic system is aroused by the invested cognitive effort and correspondingly, the pupil dilates^20^. For this reason, pupil size has been regarded as an effective proxy for neural activity^21^ and an index of cognitive effort^22–24^. In the present investigation, we adopted pupillometric methods to shed light on the unresolved question of whether the observed differences in cognitive efficiency between faster and slower performers are simply a matter of degree or represent qualitatively different patterns of activation of cognitive resources over time as proposed by Gray et al.^16^.

Another possible explanation for the inconsistent results regarding the activation-performance relationship might be the diverse range of laboratory tasks that have been employed in different investigations (e.g., CPT^16^; Tower-of-London^25^; Backward digit-span^17^), each measuring different cognitive mechanisms^15^. Rather than using the somewhat contrived laboratory tasks employed in previous research, our investigation focused on cognitive efficiency in word reading –an essential and near-universally learned skill in constant use in literate cultures. A common theme in the developmental literature on word reading is that efficient word recognition is not only fast^26–29^, but involves minimal effort^30–35^. Using pupillometry, we recently confirmed this assumption and also found that cognitive effort in reading is task-dependent^36^. Both elementary school children and skilled adult readers allocated more cognitive resources when reading unfamiliar letter strings compared to familiar words, as indexed by multiple measures of pupil dilation.

Here, we present further analyses of the data from our previous study^36^ with the aim of comparing both developmental (cross-age) and individual (within-age) differences in cognitive efficiency by measuring the cognitive effort invested in word recognition. Our first research question was whether developmental differences in cognitive efficiency between skilled adult readers (university students) and school-aged readers (4th-6th graders) are reflected in the cognitive effort involved in word recognition as measured by changes in pupil size. Since this is, to the best of our knowledge, the first study using pupillometry to examine developmental and individual differences in effort and efficiency in word recognition, we had no prior research literature on which to base our predictions. However, it seems reasonable to entertain at least two alternative hypotheses. In accordance with the neural efficiency theory^11^, the first hypothesis might be labeled the *quantitative* hypothesis. This hypothesis proposes, as noted above, that differences in efficiency are a matter of degree, that is, both skilled adult readers and school-aged readers will show the same pattern of dynamic changes in pupil dilation over time, differing only in the overall elevation (higher or lower) of these changes. According to this scenario, we predicted that skilled adult readers would demonstrate relatively less pupil dilation, reflecting lower levels of cognitive effort compared to school-aged readers due to their superior word recognition skill. Thus, cognitive efficiency in word recognition among children would be reflected in greater effort (as indicated by larger pupillary responses) compared to skilled adult readers. Alternatively, *qualitatively* distinct patterns of pupil dilation might emerge in the course of reading a word. That is, the dynamic or temporal pattern of changes in pupil dilation may differ over the course of an individual trial even though the overall amount of cognitive resources might not be significantly different^37,38^.

We also addressed the intriguing question regarding the relationship between neural activation and individual differences in performance by looking at *within-age* differences in cognitive efficiency comparing slower-performing readers to faster-performing readers. Here again, we consider both *quantitative* and *qualitative* hypotheses. According to the *quantitative* hypothesis, there will be a significant difference in the overall amount of pupil response, reflecting more effort among the slower-performers. However, according to the *qualitative* hypothesis, we would anticipate that faster performers would show a more rapid increase in brain activity when reading a word.

## Method

### Participants

#### Developmental differences

Using the data from the participants in the Shechter and Share study^36^, we compared differences between the two age-groups: the adult sample of 34 university students (27 females; mean age 27) and the school-aged sample (19 females; mean age 10) from the fourth to the sixth grades (10 fourth graders, 11 fifth graders, and 13 sixth graders). Sample size for each age-group was determined based on a prior power analysis using G*Power Version 3^39^ with power set at 0.80, alpha of 0.01, and an intermediate effect size (*f*) of 0.25. All participants were native Hebrew speakers, with no reported past or present reading difficulties or attentional deficits and normal or corrected-to-normal vision (for full details, see Shechter and Share^36^).The Ethics Committee of the Faculty of Education of the University of Haifa approved the experimental protocol (Approval no.18/427) and all relevant guidelines and regulations were adhered to in conducting this study. Each adult and each participating child’s legal guardian signed a voluntary informed consent form prior to the experiment.

#### Individual (within-age) differences

For the purposes of this analysis, each age-group was divided into subgroups on the basis of their decoding speed as follows: Response times for pseudowords pronounced correctly were averaged for each participant. Response times greater than two standard deviations above or below the participant mean were excluded. Among the adult sample, we compared the 10 fastest readers (8 females; mean age 29) to the 10 slowest readers (7 females; mean age 26). In the younger sample, the 10 fastest readers (5 females; mean age 11), included two 4th graders, three 5th graders, and five 6th graders. The 10 slowest readers (5 females; mean age 10.5), included three 4th graders, three 5th graders, and four 6th graders.

### Design

This was a between-subjects study with two levels of word lexicality/familiarity: unfamiliar letter-strings (pseudowords) and familiar (real) words. This experiment contained four blocks, each block containing 40 target stimuli: 20 pseudowords and 20 real words. The two conditions of interest here were identical and comprised 80 (20 X 4) randomized items (i.e., 160 target stimuli). Yoked pairs of familiar and unfamiliar (pseudoword) letter strings (e.g., לֶשֶׁג /sheleg/ ‘snow' שֶׁלֶג /lesheg/ pseudoword) were matched phonologically, morpho-phonologically, for length and luminance. Yoked pairs were separated by an intervening block (blocks 1 and 3 or 2 and 4).

For further details concerning the Design, Stimuli, Procedure and Apparatus see Shechter and Share^36^.

### Stimuli

The target list contained 80 word-pseudoword pairs. Each block contained several filler words (20 items for the older sample and 10 fillers for the younger sample). From a viewing distance of 57 cm, the target stimuli subtended a visual angle of 1.11° to 1.61° for height and 2.21° to 4.82° for width, and filler words subtended a visual angle of 1.00° to 1.61° for height and 1.31° to 5.82° for width. All stimuli were centered and presented in white text (RGB = 255, 255, 255) on a gray background (RGB = 128, 128, 128).

### Procedure

The data were collected in a dimly illuminated sound-reduced room at the Edmond J. Safra Brain Research Center for the Study of Learning Disabilities at the University of Haifa. Participants were asked to read aloud the displayed word which disappeared automatically. The trial sequence was the same for the adults and children except for a longer duration of stimulus presentation for the younger sample, 4700 ms instead of 3300 ms. Pronunciation onset latencies were recorded by a voice key and reading errors were manually documented by the tester.

### Apparatus

Pupillometry data were recorded with an EyeLink 1000 Plus (SR Research, Ontario, Canada), with a sampling rate of 1000 Hz. To maintain an accurate measurement of pupil size, calibration and validation preceded each block and the participants were required to keep their eyes fixed on the center of the screen during the entire session without shifting their gaze position.

### Pre-processing of pupillometry data

Pupil data were processed using the CHAP software^40^. First, pupil data were extracted from the EyeLink (pupil size in arbitrary units). Then, we removed outlier cases with Z-scores larger than 2.5. Z-scores were calculated based on the mean and standard deviation for pupil dilation for each trial. Next, we calculated the percent of outlier values for each participant in each trial and excluded from analysis trials with more than 20% missing values. We also excluded trials with incorrect or missing responses. We defined a minimum number of 20 valid trials for each condition, so no participant was excluded from the analysis. Next, we detected eye-blinks by using Hershman, Henik and Cohen’s^41^ algorithm and filled missing values by using a linear interpolation^37^. This pre-processing of pupil data eliminated 14.3% of trials on average. The exclusion rate in each condition in each group is presented in Supplementary material.

For the analysis of *quantitative* differences, the time course of pupil dilation was aligned with the onset of the stimulus and trimmed 4800 ms after stimulus onset. For the analysis of *qualitative* differences, time courses were aligned with the onset of the stimulus and divided by the baseline which was defined as the average pupil size 500 ms before the stimulus onset. This time window was defined on the basis of the typical time course of pupil-size changes (which are very different from standard cognitive-behavioral measures such as reaction time e.g.,^42,43^). In addition, taking into consideration a relatively slow return of pupil size to baseline after the main dilation^37,44,45^, the time window was extended accordingly.

## Results

### Developmental differences

**Figure 1:**
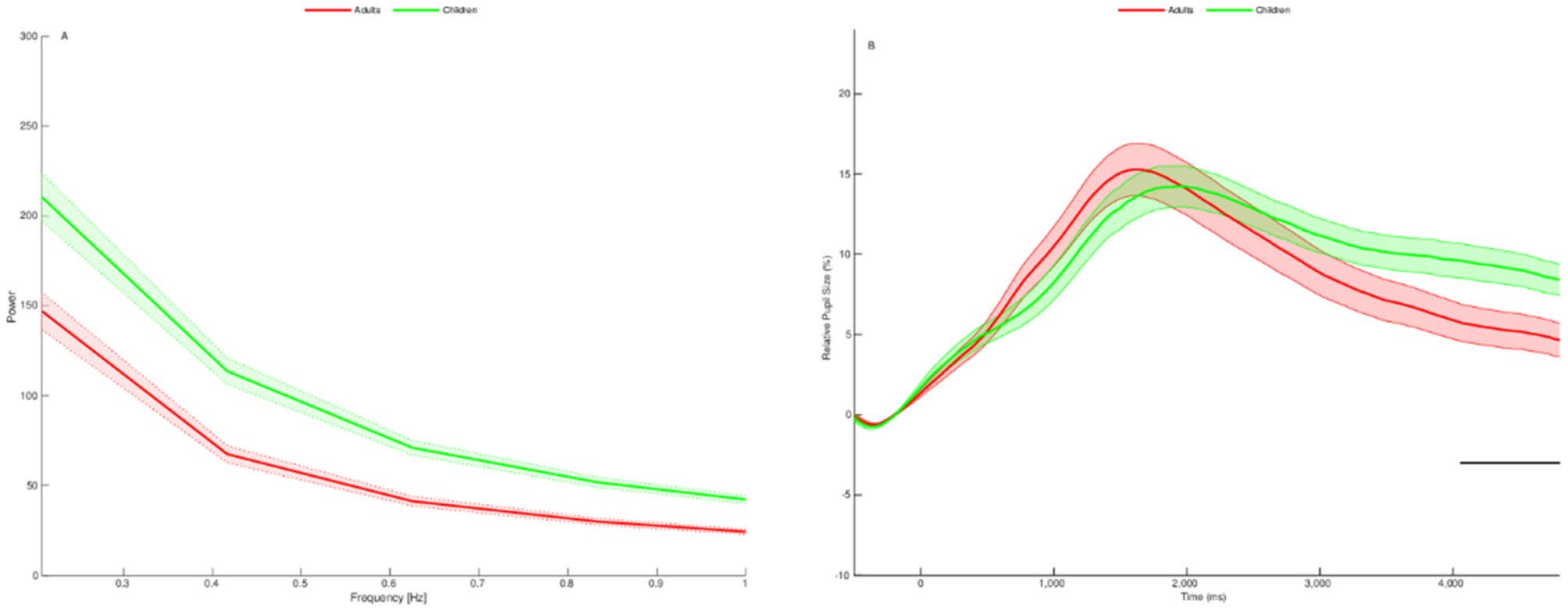
Developmental differences in pupil responses to word reading (real words and pseudowords) between adults and children. (**A**) Power as a function of frequency (smaller than 1 Hz) (**B**) Relative changes in pupil size from stimulus onset (Time 0) to 4800 ms. The shaded areas depict standard errors of the mean.

In order to test the hypothesis that the differences between more skilled readers (adults) and less skilled readers (children) is merely a matter of degree—our *quantitative* hypothesis, we examined the differences between pupil response fluctuations that were smaller than 1 Hz (i.e., wavelengths that were larger than 1 s). These fluctuations have been proposed as a measure of mental effort^41^. Using Fourier transform, we converted the time window between stimulus onset and 4,800 ms post stimulus onset from the time space into the frequency space. Then, mean power of frequencies that were smaller than 1 Hz at each measured frequency was calculated separately for each condition (i.e., real words and pseudowords) for each participant for each group. We then defined, for each participant, the mean power as the mean power of both real words and pseudowords. Finally, an independent sample t-test between the groups was conducted. Consistent with the quantitative hypothesis, the power of the low frequencies of the children was larger compared to those of the adults ($t \left( {66} \right) = 5.148,p < .001,BF_{10} = 5,770.149, Cohen^{^{\prime}} s\:d =$ 1.248) (Fig. 1A). (Since the pupil has a relatively large signal-to-noise ratio, the use of *t*-tests in which participant means are averaged across items has been recommended as the appropriate statistical approach^43,48^).

Next, for testing our *qualitative* hypothesis, we examined the temporal differences^44^ between the adults and the children using Hershman and Henik's^37^ approach. Specifically, we ran a series of Bayesian independent sample t-tests between the groups. Each group was defined by the average pupil size of both real words and pseudowords. As anticipated, more pupil dilation was observed for developing readers compared to skilled adult readers but, consistent with the qualitative hypothesis, this difference was not uniform across the entire time window. This difference in pupil dilation began around 4,050 ms post stimulus onset and remained until the end of the trial (Fig. 1B).

Note that the stimuli were presented in white and remained visible for a longer duration for the school-aged sample (4700 ms compared to 3300 ms for the adult sample). In other words, the children were presented with bright stimuli for a longer time. Therefore, one would expect *less* dilation for this group simply because pupils constrict more in brighter conditions^46^. This means that the greater dilation among children cannot be attributed to light intensity but to their cognitive response to the stimuli.

Our results confirmed the existence of developmental differences in cognitive efficiency between school-aged readers and skilled adult readers, as reflected by the cognitive effort invested in word reading. First, *quantitative* differences were observed between the two age-groups, with children demonstrating a greater degree of cognitive effort when reading a word compared to skilled adults. Second, temporal examination revealed *qualitatively* distinct patterns of pupil responses between the groups in which relative pupil size remained larger among children but not among adults at the end of the process.

### Individual (within-age) differences

**Figure 2:**
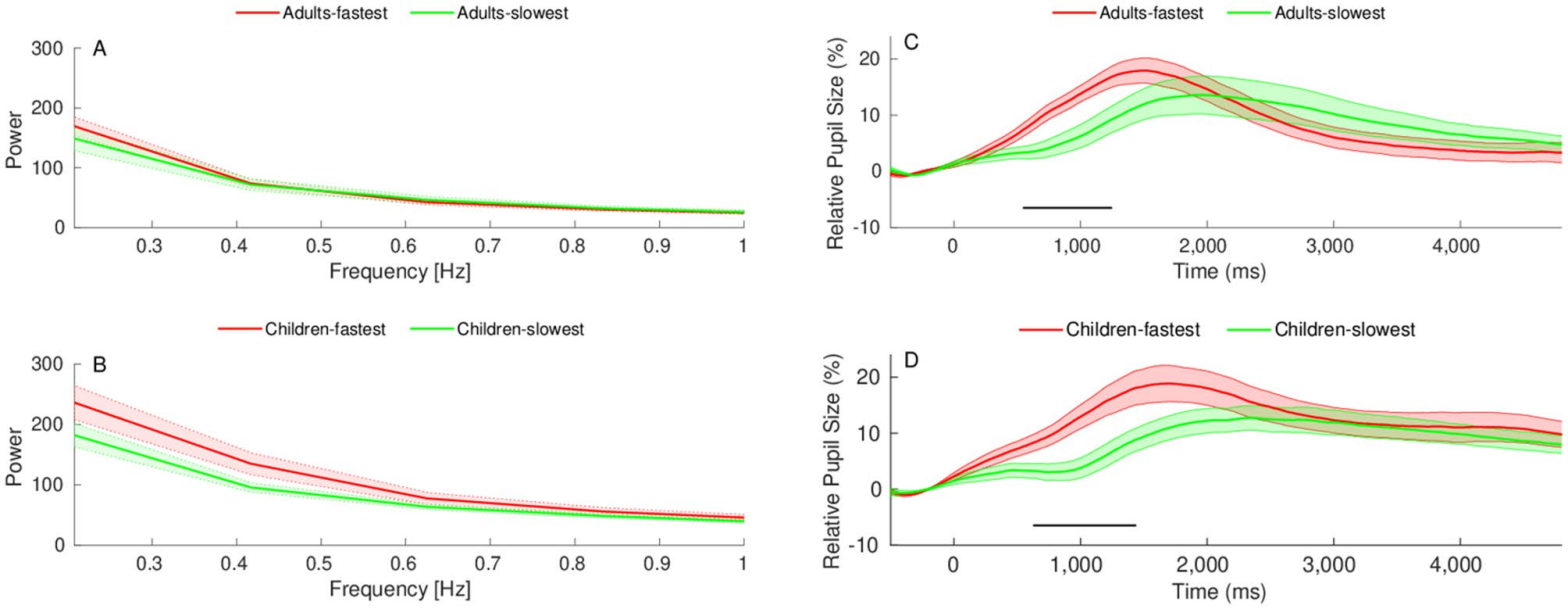
Individual differences in pupil responses to word reading (real words and pseudowords) between faster readers and slower readers. (**A**,**B**) Power as a function of frequency (smaller than 1 Hz) among the (**A**) adult sample. (**B**) school-aged sample. (**C**,**(D**) Relative changes in pupil size from stimulus onset (Time 0) to 4800 ms among the (**C**) adult sample. (**D**) school-aged sample. The shaded areas depict standard errors of the mean.

For each age group, we then examined *quantitative* and *qualitative* differences between the 10 fastest readers and the 10 slowest readers. As before, we tested the *quantitative* hypothesis by comparing pupil response fluctuations that were smaller than 1 Hz (i.e., wavelengths that were larger than 1 s). An independent sample t-test conducted for each age group separately revealed no differences between the fastest readers and the slowest readers (${\text{adults}}:{ }t \left( {18} \right) = 0.315,\,\,p = .757,\,\,BF_{10} = 0.412,\,\, Cohen^{^{\prime}} s \,\,d = 0.141$; ${\text{children}}:{ }t \left( {18} \right) = 1.646,p = .117,BF_{10} = 0.995, Cohen^{^{\prime}} s\:d = 0.736$) (Fig. 2A and B). This indicated that faster and slower readers in each age group invested similar amounts of overall cognitive effort in word reading, but, as we next show, there were significant qualitative differences between the subgroups.

In order to test our *qualitative* hypothesis, we ran a series of Bayesian independent sample t-tests between the groups. Each group was defined by the average pupil size of both real words and pseudowords. Consistent with our *qualitative* hypothesis and the results of the developmental analysis, subgroup differences were not uniform across the time window. In the adult sample, significantly greater pupil dilation was observed for the fastest readers over a 700 ms interval between 560 and 1260 ms (Fig. 2C). This pattern of results was replicated among our younger readers. That is, significantly greater dilation was observed for the fastest readers compared to the slowest readers over a very similar time interval, from 640 ms post stimulus onset through to 1430 ms post stimulus onset (Fig. 2D).

In both age groups, we found the same pattern of results, thus our findings support the *qualitative* hypothesis and reject the *quantitative* one. That is, while the fastest readers and the slowest readers invested a similar degree of overall cognitive effort in word reading, the fastest readers demonstrated accelerated pupillary responses compared to the slowest readers.

## Discussion

Throughout the history of modern psychology, the neural basis of cognitive performance, and particularly its efficiency, has been assumed to be an essential determinant of individual differences in a wide range of human behaviors. In the current investigation, we examined one aspect of cognitive efficiency—cognitive effort^5,6^. To this end, we used pupillometry as a proxy for neural activity^21^ and an indicator of cognitive effort^22–24^.

Our investigation of cognitive efficiency also wished to avoid the typical contrived laboratory tasks that limit generalizability to the real world by focusing on the allocation of effort in reading – an essential and ubiquitous skill. Specifically, this investigation sought to test the theoretical premise that efficient word recognition involves less effort^30–35^. We examined both developmental (cross-age) and individual (within-age) differences in cognitive efficiency by measuring the cognitive effort invested in word recognition among skilled adult readers (university students) and school-aged readers (4th-6th graders) and between subgroups of faster and slower readers within each age group.

Our findings provided clear evidence for the existence of developmental differences in cognitive efficiency between readers in accordance with their reading proficiency, both in *quantitative* and *qualitative* terms. Quantitatively, we found that skilled adult readers showed lower levels of cognitive effort when reading a word compared to school-aged readers. That is, less pupil dilation was observed among the adult sample, reflecting lower levels of invested effort in word reading. This pattern of results supports the neural efficiency theory^11^, in which skilled performers are assumed to demonstrate overall less activation than less skilled performers. Qualitatively, their superior word recognition skill was manifested in differences in the temporal pattern of changes in pupil dilation over time as well. Pupil size remained larger among children at the end of the word reading process, a pattern which might point to their sustained effort in the course of reading a word compared to the adults. This observation provides a window into the developmental nature of reading skill. According to our findings, developing readers invest more cognitive effort in word recognition compared to highly skilled readers, as reflected both by the amount of the resources^11^ and the speed with which the nervous system responds^9,10^.

The within-age examination of differences in cognitive efficiency based on reading speed yielded a similar pattern of results among adults and school-aged readers alike. In *quantitative* terms, we found no difference in the overall cognitive effort invested in word recognition between the fastest readers and the slowest readers. However, in *qualitative* terms, the fastest readers showed accelerated pupillary responses compared to the slowest readers. These findings contradict previous studies that have reported quantitative differences in neural activity between faster performers and slower performers^12–14^, although it must be acknowledged that different tasks were employed. However, in accordance with Gray et al.,^16^ we observed increased brain activation at the beginning of the reading process among the faster readers, suggesting that they maximized their resource allocation at the initial phase of word recognition.

Summarizing these findings, we conclude that the faster readers and the slower readers within the same age cohort deploy the same amount of cognitive resources while reading a word but differ in the speed of their neural processing^9,10^. We hasten to point out that our participants were all typical readers only, thus the question of cognitive efficiency among struggling or disabled readers remains a subject for further inquiry.

Our findings have the potential to open up new avenues of research capable of providing a deeper understanding and better operationalization of the much-debated concepts of fluency, automaticity, and cognitive efficiency. Pupillometry offers reading researchers a more sensitive moment-by-moment glimpse into the dynamics of word recognition (including developmental, inter-individual, and intra-individual variation) that goes beyond the standard measures of skill growth such as reading accuracy and rate or some combination of these two (such as words correctly read per minute). Despite the fact that terms such as “effortless” and “effortful” are constantly used to describe and even define non-proficient and proficient reading respectively [see, e.g., the DSM-5 definition of *specific learning disability in reading* (p.66)^47^], no objective measures of effort exist. And because learning to read is a quintessential case of skill learning, pupillometry has potentially far-reaching application to all domains of skill learning.

## Supplementary Information

Supplementary Information.

## Data Availability

Data for all experiments are publicly available on OSF at https://osf.io/hk4yq/.
