# Supplementary material for: A pupillometric study of developmental and individual differences in cognitive effort in visual word recognition
Source: Sci Rep. 2022 Jun 24;12:10764. doi: 10.1038/s41598-022-14536-9 (PMC9232497; doi:10.1038/s41598-022-14536-9)
Supplement: Supplementary file 1 — Supplementary Information. [file 41598_2022_14536_MOESM1_ESM.docx]

**Supplementary material**

Exclusion rate in each condition for the developmental analysis. We excluded from the analysis incorrect trials or trials with more than 20% of missing values.

|  | Adults | | Children | |
| --- | --- | --- | --- | --- |
|  | Real words | Pseudowords | Real words | Pseudowords |
| Correct responses (%) | 97.9 | 89.7 | 92.0 | 76.7 |
| Valid trials (%) | 94.0 | 87.2 | 87.7 | 73.8 |
